# Development of a novel prognostic model for mantle cell lymphoma based on quantitative detection of CD3 by quantitative dot blot

**DOI:** 10.3389/fonc.2025.1595572

**Published:** 2025-09-10

**Authors:** Ning Zhu, Yunjun Wang, Jiajia Hu, Xiaoyan Lin, Cuijuan Zhang, Fangrong Tang, Guohua Yu

**Affiliations:** ^1^ Department of Pathology, College of Basic Medical Sciences, Binzhou Medical University, Yantai, Shandong, China; ^2^ Department of Pathology, Yantai Yuhuangding Hospital Affiliated Qingdao University, Yantai, Shandong, China; ^3^ Department of Pathology, College of Basic Medical Sciences, Southwest Medical University, Luzhou, Sichuan, China; ^4^ Department of Pathology, Shandong Provincial Hospital Affiliated to Shandong First Medical University, Jinan, Shandong, China; ^5^ Department of Pathology, Qilu Hospital, Cheeloo College of Medicine, Shandong University, Jinan, Shandong, China; ^6^ Yantai Quanticision Diagnostics, Inc., a Division of Quanticision Diagnostics, Inc., of USA, Yantai, Shandong, China

**Keywords:** CD3, mantle cell lymphoma international prognostic index, mantle cell lymphoma, quantitative dot blot, immunohistochemistry

## Abstract

**Background:**

The Mantle Cell Lymphoma International Prognostic Index (MIPI) is the standard risk stratification model, but it primarily relies on clinical parameters and does not incorporate molecular markers. Studies suggest that CD3+ T cells, as a key component of the tumor microenvironment (TME), play a crucial role in mantle cell lymphoma (MCL) progression and prognosis. However, conventional immunohistochemistry (IHC) has limitations in quantifying CD3 expression due to its subjectivity and variability. Quantitative Dot Blot (QDB) is an emerging high-throughput protein quantification technique that allows for precise measurement of CD3+ T cells. This study aimed to evaluate the prognostic significance of CD3+ T cells quantified using QDB and IHC in MCL patients and to introduce the MIPI/CD3 model to enhance risk stratification and improve prognostic accuracy.

**Methods:**

A retrospective analysis of 120 newly diagnosed MCL patients from four hospitals between 2008 and 2020. The CD3 expression was assessed using both IHC and QDB. Patients were classified into CD3^low^ and CD3^high^ groups based on an optimal cutoff value. MIPI and MIPI-c scores were calculated, and a novel MIPI/CD3 model was developed by integrating QDB-based CD3 quantification. Kaplan-Meier survival curves were used to evaluate overall survival (OS), and differences between groups were compared using the log-rank test. A p-value < 0.05 was considered statistically significant.

**Results:**

CD3 quantification by IHC was correlated with OS (p=0.47), whereas QDB-based CD3 quantification showed a significant association with OS (p=0.0051), with the CD3^high^ group exhibiting better prognosis compared to the CD3^low^ group. The MIPI/CD3 model outperformed both the MIPI and MIPI-c models in prognostic prediction (p=0.0075) and demonstrated greater accuracy in distinguishing between low-risk and high-risk patients.

**Conclusion:**

CD3+ T cells are an important prognostic biomarker in MCL, with high expression indicating a better prognosis. Integrating CD3 into the MIPI model enhances risk stratification accuracy. Compared to traditional IHC, QDB provides a more precise and reliable method for measuring CD3+ T cells. However, further validation in larger MCL cohorts is necessary to confirm its clinical utility. Future research should integrate immune and molecular biomarkers to further refine MCL risk models and advance personalized treatment.

## Introduction

Mantle cell lymphoma (MCL) is a rare and aggressive subtype of B-cell non-Hodgkin lymphoma, with a median age of 65 years and overall survival (OS) time of 3 to 5 years (1, 2). The t (11, 14) translocation leads to Cyclin D1 overexpression, causing cell cycle disruption and further promoting the development of MCL (3). Currently, the MCL International Prognostic Index (MIPI) is the internationally recognized model for risk stratification in MCL (4). However, MIPI primarily relies on clinical parameters such as age, LDH levels, clinical stage, and white blood cell count, neglecting other potential prognostic factors like molecular markers (5–7). As it does not fully reflect the biological characteristics and complexity of the disease in MCL patients, identifying effective molecular markers for prognosis and treatment assessment is of great importance.

There is a significant interaction between tumor cells and immune cells in the tumor microenvironment (TME). Nygren et al. reported (7) that in lymph nodes and peripheral blood, both the number of CD4-positive T cells and the CD4/CD8 ratio are positively correlated with OS in MCL. CD3-positive T cells are more abundantly expressed in MCL. These CD3-positive T cells can be further categorized into CD4-positive and CD8-positive T cells. CD4-positive T cells contribute to the inhibition of tumor growth by directly secreting cytokines such as IFN-γ and TNF, which suppress tumor angiogenesis (8). Additionally, CD4-positive T cells enhance anti-tumor immune responses indirectly by activating other immune cells, such as CD8-positive T cells (8, 9).

Immunohistochemistry is inherently qualitative, and due to the subjective interpretation by pathologists, the assessment results may vary. This lack of objectivity and consistency prevents IHC from providing quantitative analysis (10, 11). In contrast to traditional IHC methods, Quantitative Dot Blot (QDB) is an emerging technique that allows for absolute quantification, enabling precise and high-throughput analysis of specific protein levels in tissues (12). To better understand the role of CD3-positive T cells in MCL, this study collected surgical specimens and clinicopathological data from 120 MCL patients. By using IHC and QDB technique to assess CD3 expression, we explored the relationship between CD3-positive T cells and prognosis, and investigated the potential of combining CD3 with the MIPI (CD3/MIPI) to improve the accuracy of the risk model.

## Patients and methods

### Patients

We conducted a retrospective analysis of MCL patients diagnosed in the Department of Pathology at Yantai Yuhuangding Hospital, Qilu Hospital, Shandong Provincial Hospital, and the Affiliated Hospital of Southwest Medical University between January 1, 2008, and September 8, 2020. All patients provided written informed consent and covering admission. All samples were confirmed as MCL by three pathologists based on morphological and immunohistochemical findings. Patients were newly diagnosed, with no prior treatment before diagnosis, and cases with uncertain diagnoses were excluded. Additionally, patient survival data was meticulously documented through follow-ups with patients or their relatives. OS was calculated from the date of initial diagnosis to the time of death.

### General reagents

All chemicals were sourced from Sinopharm Chemicals (Beijing, P.R. China). The recombinant human CD3 (223–207) protein was obtained from Cusabio (Wuhan, P.R. China). Anti-CD3 (EP41) rabbit monoclonal primary antibody was purchased from ZSGB-BIO (Beijing, P.R. China). HRP-donkey anti-Rabbit lgG secondary antibody was purchased from Jackson lmmunoresearch lab (West Grov, PA, USA). BCA protein quantification kit was purchased from Thermo Fisher Scientific Inc. (Calsband, CA, USA). QDB plates were supplied by Yantai Quanticision Diagnostics, Inc. (Yantai, P.R. China).

### IHC-based CD3 scoring

The standard streptavidin-biotin complex method was employed, using 3,3’-diaminobenzidine as the chromogen, and antibodies provided by Yantai Yuhuangding Hospital were used to stain the slides. The three pathologists responsible for IHC assessment of CD3 were blinded to the clinicopathological characteristics and outcomes. The number of CD3-positive cells was counted in ten randomly selected high-power fields at 400x magnification, and the average count was calculated.

### Preparation of FFPE tissue

Two FFPE tissue sections (2×5 µm) from each specimen were placed into 1.5 mL Eppendorf tubes, deparaffinized, and then dissolved in lysis buffer (50 mM HEPES, 137 mM NaCl, 5 mM EDTA, 1 mM MgCl_2_, 10 mM Na_2_P_2_O_7_, 1% Triton X-100, 10% glycerol). After centrifugation, the supernatant was collected, and the total protein content was measured using a BCA protein assay kit according to the manufacturer’s instructions.

### QDB analysis

The specific QDB process has been described in detail in our previous studies, with slight modifications (12). Briefly, 2 μL of FFPE tissue lysates per unit were used for QDB analysis, performed in triplicate. The loaded QDB plates were air-dried at room temperature for 1 hour and then blocked with 5% skim milk for 1 hour. The anti-CD3 antibody was diluted 1:1,000 in blocking buffer and incubated overnight at 4 °C with 100 µL per well, followed by a 4-hour incubation at room temperature with donkey anti-mouse secondary antibody. The plates were then treated with ECL reagent and quantified using a Tecan Infiniti 200 Pro microplate reader. Absolute CD3 levels were determined based on a protein standard dose-response curve. The results represent the average of three independent experiments. An experiment was considered valid if the measured levels of the target protein were within ±10% of the recorded values. Samples with fluorescence readings less than twice that of the blank were defined as undetectable and recorded as 0 in the data analysis.

### MIPI and CD3/MIPI

The original MIPI scores were evaluated for each patient. According to standard guidelines, patients were classified into different risk subgroups (13). Using the ‘surv_cutpoint’ function from the ‘survivminer’ R package, the optimal cutoff value for CD3 levels was determined, and the CD3 levels detected by the QDB and IHC methods were dichotomized. Samples with CD3 levels below the cutoff in the QDB method were assigned 1 point, while those above the cutoff were assigned 0 points. For the MIPI score, a score of 0 to 3 was assigned 1 point, 4 to 5 was assigned 2 points, and 6 to 11 was assigned 3 points. The CD3 score and the MIPI score for each case were then summed, with a total score of 1 indicating low risk, 2 indicating low-intermediate risk, 3 indicating high-intermediate risk, and 4 indicating high risk groups.

### Statistical analysis

The optimal cutoff value for CD3 levels detected by the QDB and IHC method was determined using the “surv_cutpoint” function in the “survivminer” R package. Statistical analyses were performed using R software version 4.3.3. All OS analyses were visualized using the Kaplan-Meier method and compared using the Log-rank test. A p-value of less than 0.05 was considered statistically significant.

## Results

**Figure 1:**
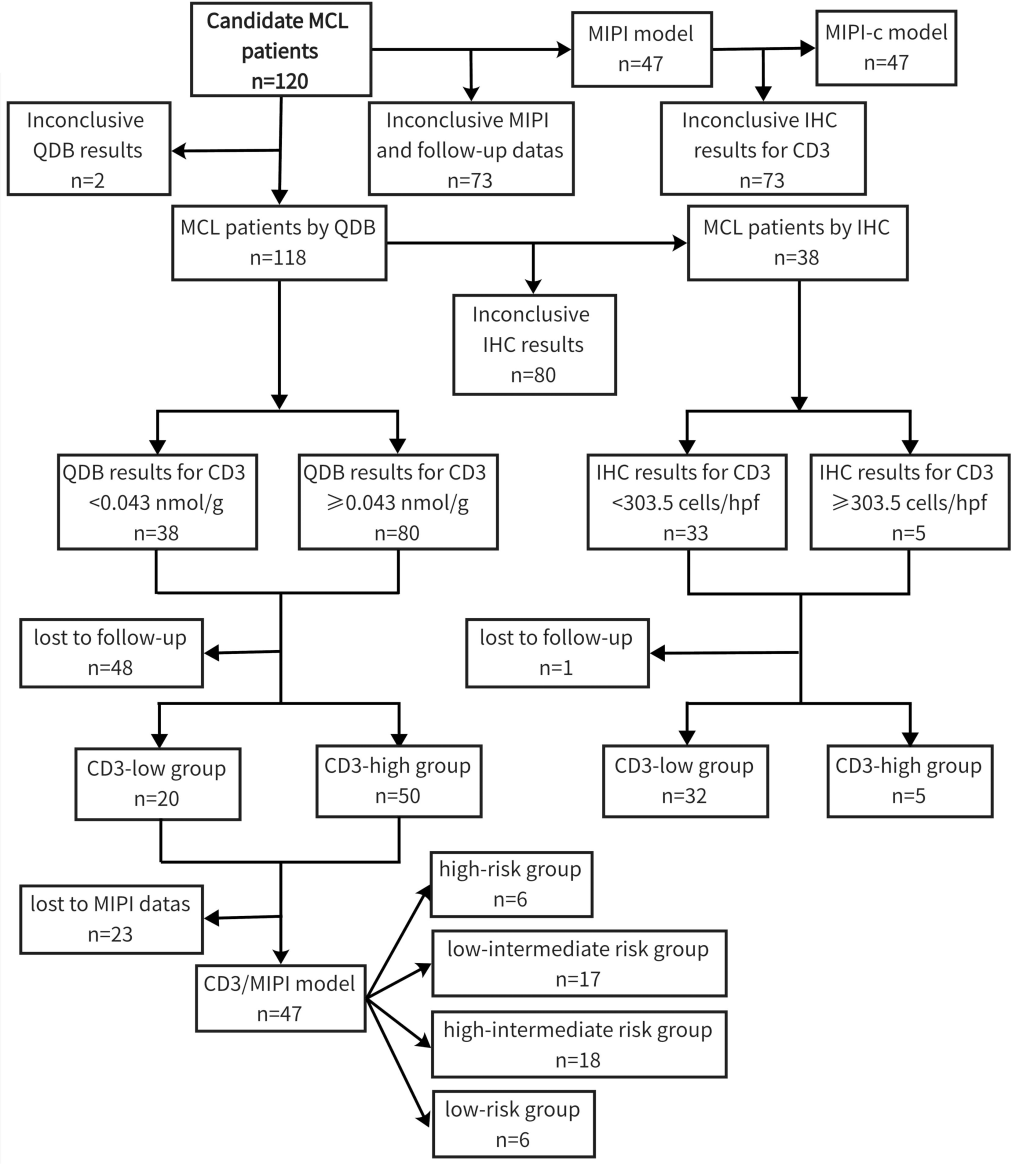
The flowchart for patient selection for the study.

Among the 120 newly diagnosed MCL patients included in the study, despite incomplete clinicopathological information, 87 were male and 32 were female, resulting in a male-to-female ratio of approximately 2.7:1. The median age was 63 years (range: 35–86 years). Follow-up records were available for 71 individuals, with a follow-up period ranging from 0 to 96 months and a median duration of 24 months. By the end of the follow-up, 42 patients had succumbed to the disease, resulting in an overall mortality rate of 59.2%. We used the “surv_cutpoint” function from the “survminer” R package to determine cutoff values of 0.042 nmol/g for CD3 quantification by the QDB method and 303.5 cells/hpf for CD3 quantification by the IHC method, categorizing patients into two groups: CD3^low^ (<0.043 nmol/g or <303.5 cells/hpf) and CD3^high^ (≥0.043 nmol/g or ≥303.5 cells/hpf). The MIPI model identified 7 cases in the low-risk group, 18 cases in the intermediate-risk group, and 22 cases in the high-risk group. Based on the CD3-QDB results and the risk stratification of the MIPI model, the CD3/MIPI model categorized 6 patients into the low-risk group, 17 patients into the low-intermediate risk group, 18 patients into the high-intermediate risk group, and 6 patients into the high-risk group (**Table 1**). The study flowchart can be found in **Figure 1**.

**Table 1:** Log-rank testing for Clinicopathological characteristics of patients.

Variables	Number (%)	p value
CD3-IHC (cells/hpf)		0.47
<303.5	33 (86.5)	
≥303.5	5 (13.5)	
CD3-QDB (nmol/g)		0.0051
<0.043	20 (27.1)	
≥0.043	50 (72.9)	
MIPI		0.048
Low risk	7 (16.7)	
Intermediate risk	18 (37.5)	
High risk	22 (45.8)	
MIPI/CD3		0.0075
Low risk	6 (12.5)	
Low-Intermediate risk	17 (37.5)	
High-Intermediate risk	18 (39.6)	
High risk	6 (10.4)	
MIPI-c		0.08
Low risk	4 (8.7)	
Low-Intermediate risk	10 (23.9)	
High-Intermediate risk	11 (23.9)	
High risk	21 (43.5)	

**Figure 2:**
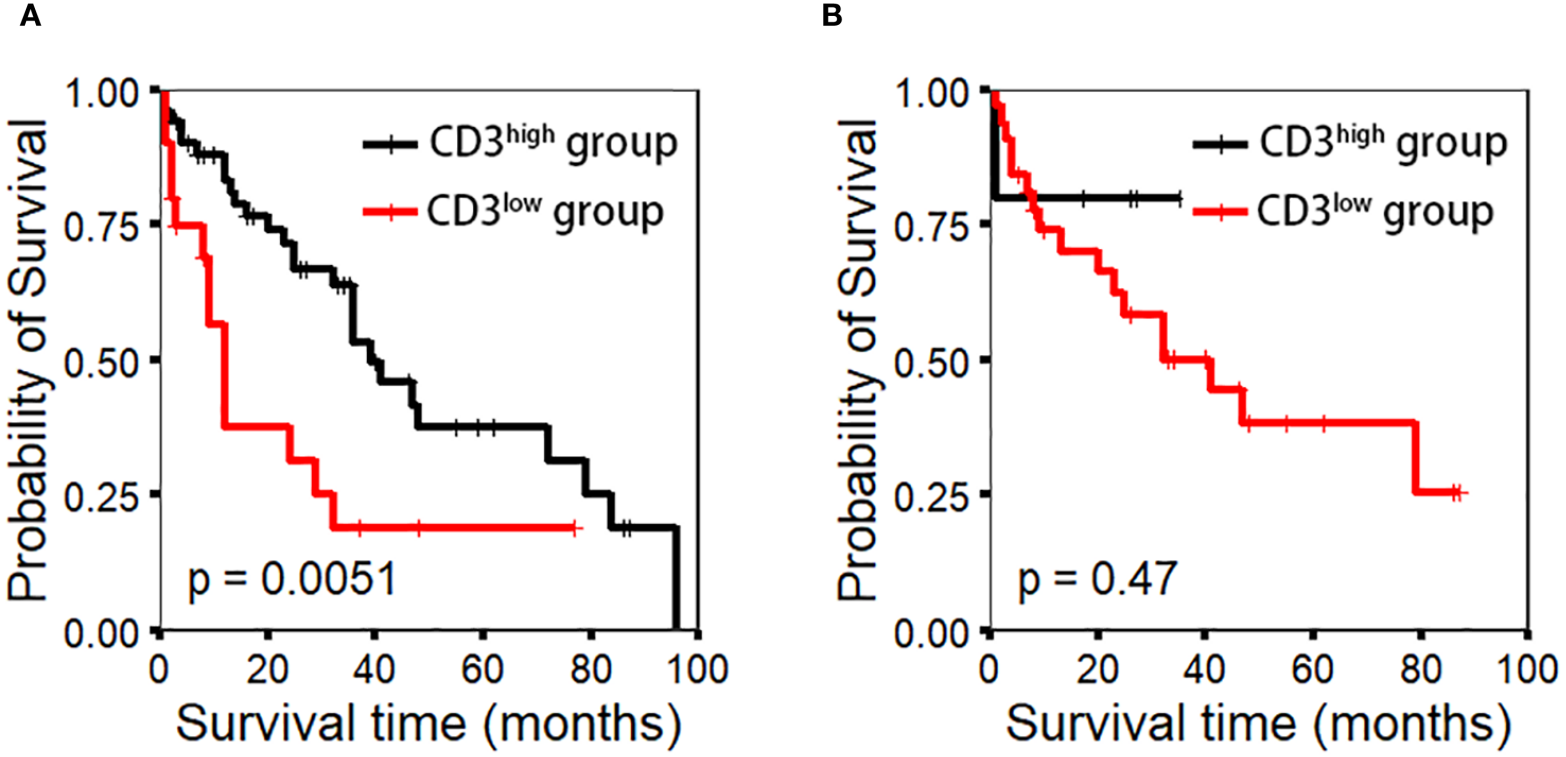
CD3 expression using the IHC method was evaluated through manual counting under a microscope, while the QDB method was used for precise quantification. Appropriate cutoff values (0.043 nmol/g for QDB and 303.5 cells/hpf for IHC) were determined to assess patient prognosis. **(A)** CD3 expression by the QDB method (p-value=0.0051). **(B)** CD3 expression by the IHC method (p-value=0.47).

**Figure 3:**
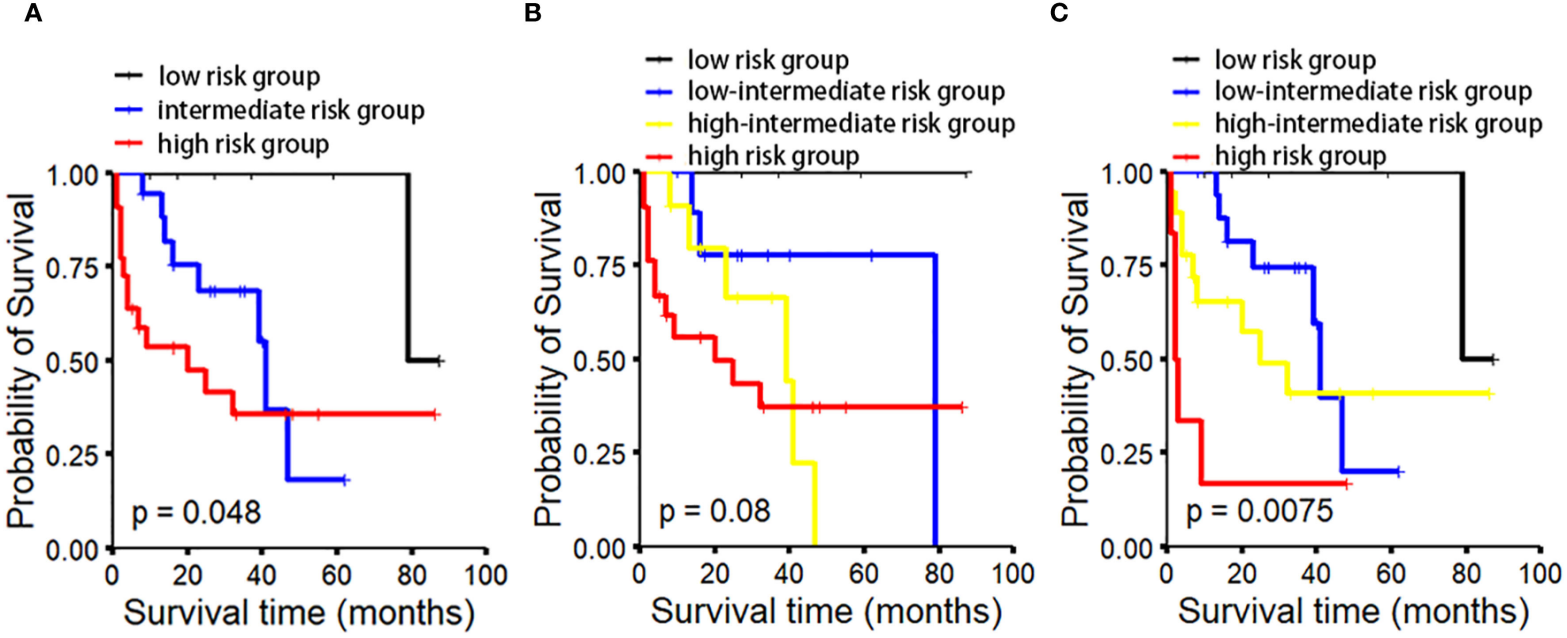
OS analysis in MCL patients based on different prognostic models. OS analysis based on MIPI/CD3 **(C)** demonstrates superior performance in distinguishing between the low-risk and high-risk groups compared to MIPI **(A)** and MIPI-c **(B)**.

Quantitative analysis of CD3-positive cells using IHC with manual counting revealed no statistically significant difference in OS between the CD3^low^ and CD3^high^ groups (p=0.47). Subsequently, we performed absolute quantification of CD3 using the QDB method, which demonstrated a statistically significant difference in OS (p=0.0051) (**Figure 2**). The analysis revealed a statistically significant difference in OS among the risk groups of MIPI (p=0.048). However, the MIPI-c model demonstrated limited effectiveness in distinguishing between different risk groups (p=0.08). We found that, compared to the MIPI and MIPI-c models, the CD3/MIPI model demonstrated statistically significant differences in survival across different groups (p=0.0075) and exhibited greater accuracy in identifying both low-risk and high-risk patients (**Figure 3**).

## Discussion

As research on MCL has deepened, the concept of the TME has garnered increasing attention, with its role in disease progression and prognosis becoming more apparent (14–16). The TME consists of tumor cells, microvasculature, stromal cells, and a small number of infiltrating immune cells, such as T lymphocytes (17). T cells within the TME may affect patient prognosis. Studies have shown that CD3+ T cell infiltration is associated with better outcomes, a finding already confirmed in gastric cancer, gastrointestinal pancreatic neuroendocrine tumors, glioblastoma, and colorectal cancer (18–21). CD3+ T cells recognize tumor-associated antigens on the surface of tumor cells, release cytokines (such as interferon-gamma), and directly kill tumor cells, thereby inhibiting tumor growth. Furthermore, CD3+ T cells reduce tumor recurrence through an immune memory mechanism (19, 20). A higher level of CD3+ tumor-infiltrating lymphocytes (TILs) indicates a stronger anti-tumor immune response, which correlates with better survival rates and treatment outcomes. Moreover, in patients with PD-L1 expression, the combination of CD3+ TILs may suggest a higher sensitivity to immune checkpoint inhibitors, such as PD-1/PD-L1 inhibitors, potentially leading to improved survival (19, 21).

However, research on CD3+ T cells in lymphoma is relatively limited. Nygren et al. reported that higher levels of CD3+ and CD4+ T cells, as well as a higher CD4/CD8 ratio, are closely associated with better prognosis, a finding that has been confirmed in MCL (7). CD4+ T cells enhance antitumor responses through multiple mechanisms. They secrete cytokines such as IL-2 and IFN-γ, deliver co-stimulatory signals, facilitate efficient antigen presentation by dendritic cells, and promote antibody production by B cells (22). Experimental studies have demonstrated that CD4+ T cells play a critical role in sustaining antitumor immunity by maintaining the function of CD8+ cytotoxic T lymphocytes, preventing their activation-induced cell death, and promoting the establishment and maintenance of long-term immunological memory (23, 24). Assis-Mendonça et al. conducted a detailed analysis of the TME composition in 88 MCL tumor samples and found that higher FOXP3/CD3 and CD8/CD3 ratios were associated with worse event-free survival. Higher CD8/CD3 ratios were more commonly observed in MIPI high-risk patients (15). Although the precise mechanisms by which CD3+ T lymphocytes in the TME inhibit tumor progression are not fully understood, the available evidence suggests that CD3+ T cells may serve as an indicator of prognosis and guide early chemotherapy interventions in clinical practice. Lokhande et al. demonstrated that higher CD3+ T cell infiltration in MCL is associated with better survival. Through spatial and molecular profiling, they found that CD8+ cytotoxic T cells in tumor-rich regions expressed effector molecules such as PRF1, GZMK, and TNF, indicating preserved cytotoxic function. Additionally, tumor cells in these regions upregulated genes such as IL7R, CD80, and NLRC5, which are involved in T cell survival, co-stimulatory signaling, and antigen presentation, thereby supporting T cell recruitment, activation, and sustained immune responses (25).

QDB is a novel, precise and high-throughput method for protein quantification, with broad applications in clinical biomarker detection (12). QDB has been applied in research on breast cancer, gastric cancer, thyroid cancer, and myotonic dystrophy myoblasts (26–38). Currently, there are few reports on the application of QDB in MCL research. Yang et al. conducted a quantitative analysis of Cyclin D1 using the QDB technique and found that, with a cutoff value of 0.46 nmol/g, MCL with high Cyclin D1 expression had longer survival, thereby confirming the crucial role of Cyclin D1 in the prognostic evaluation of MCL (31). Using QDB technology, we conducted quantitative detection of CD3 in tissue samples and analyzed the correlation between CD3 levels and patient prognosis. The results indicated that CD3+ T cells in the TME of MCL patients serve as a favorable prognostic factor, validating their potential role in tumor resistance. Compared to traditional IHC, QDB not only overcomes the limitations of IHC in precise quantification but also avoids the common false-positive issues associated with enzyme-linked immunosorbent assay (12, 26, 30, 35, 37). Currently, MIPI and MIPI-c are important prognostic tools for MCL. To further enhance the accuracy of risk stratification, we integrated the absolute quantification of CD3 with the MIPI score for survival analysis. The results demonstrated that, compared to MIPI or MIPI-c alone, the MIPI/CD3 model exhibited superior performance in prognostic evaluation. Our study confirmed the prognostic value of CD3+ T cells in the TME of MCL and established the MIPI/CD3 model to enhance the accuracy of patient risk stratification. However, to further validate our findings, a larger sample size is required to strengthen the robustness of the results.

While this study provides valuable insights, it has some limitations. The sample size was relatively small, and only 71 out of 120 MCL patients had complete follow-up data, which may reduce the statistical power and introduce selection bias. To improve the reliability and generalizability of the MIPI/CD3 model, we plan to conduct external validation in larger, multi-center prospective studies. At the same time, we will continue to advance the clinical use of QDB technology as a standardized tool for biomarker detection in MCL.

## Conclusions

This study confirmed the prognostic significance of CD3+ T cells, as quantified using the QDB method, in MCL and established the MIPI/CD3 model to enhance risk stratification. Compared to MIPI and MIPI-c, MIPI/CD3 demonstrated superior prognostic performance. Additionally, QDB outperformed traditional IHC in the absolute quantification of CD3, but its clinical utility requires further validation in larger cohorts.

## Data Availability

The raw data supporting the conclusions of this article will be made available by the authors, without undue reservation.
